# Blood lead level evaluation in children presenting with chronic constipation in Tehran-Iran: a cross-sectional study

**DOI:** 10.1038/s41598-023-29487-y

**Published:** 2023-02-09

**Authors:** Nasim Zamani, Amirhossein Hosseini, Fariba Farnaghi, Aliakbar Sayyari, Narges Gholami, Farid Imanzadeh, Seyed Kaveh Hadeiy, Mahmoud Hajipour, Amir Salimi, Scott Philips, Hossein Hassanian-Moghaddam

**Affiliations:** 1 Social Determinants of Health Research Center, Shahid Beheshti University of Medical Sciences, Tehran, Iran; 2 Allianz Research Institute, Westminster, CA, USA; 3 Pediatric Gastroenterology, Hepatology and Nutrition Research Center, Research Institute for Children’s Health, Shahid Beheshti University of Medical Sciences, Tehran, Iran; 4 Department of Pediatrics, Loghman Hakim Hospital, School of Medicine, Shahid Beheshti University of Medical Sciences, Tehran, Iran; 5 School of Medicine, Shahid Beheshti University of Medical Sciences, Tehran, Iran; 6 University of Colorado Anschutz Medical Campus, Rocky Mountain Poison and Drug Safety, Denver, CO USA; 7 Washington Poison Center, Seattle, WA USA; 8 Department of Clinical Toxicology, Shohada-e-Tajrish Hospital, School of Medicine, Shahid Beheshti University of Medical Sciences, Tehran, Iran

**Keywords:** Health care, Risk factors, Signs and symptoms

## Abstract

Constipation is a common reason for children seeking medical care worldwide. Abdominal complaints and constipation are also common in lead-poisoned children. This study evaluates the prevalence of abnormal blood lead levels (BLL) among pediatric and adolescent patients and examines the association of constipation with elevated BLL. This was a prospective data collection of patients younger than 18 years old with the chief complaint of constipation seen in the Mofid Children’s Hospital gastroenterology clinic and Loghman Hakim pediatric and pediatric gastroenterology clinics were eligible for enrollment in this study. Constipation was defined as infrequent or difficult defecation according to ROME IV criteria lasting 2 months or more. BLL was measured with a fresh capillary whole blood capillary sample. The LeadCare II device assays BLL using an electrochemical technique (anodic stripping voltammetry). A total of 237 patients were enrolled in the study. 122 (51.48%) were female and 115 (48.52%) were male. About one fifth of patients (49; 20.67%) had BLL ≥ 5 µg/dL. The mean BLL in the sample was 3.51 µg/dL. Abdominal pain was the most common symptom accompanying constipation (134; 56%). Multivariate analysis found endoscopic evaluation (P values 0.024, OR 3.646, 95% CI 1.189–11.178), muscle pain (P values 0.020, OR 24.74, 95% CI 1.67–365.83), and maternal education (P values 0.02, OR 4.45, 95% CI 1.27–15.57) with significant differences in groups of patients with normal and elevated BLL. Elevated BLL necessitates an assessment and plans to reduce childhood lead exposure. BLL screening in childhood constipation with refractory chronic abdominal pain may also eradicate the need for invasive procedures like endoscopic evaluation.

## Introduction

Constipation is recognized as infrequent or difficult defecation diagnosed according to ROME IV criteria^1^. Constipation is a common complaint in pediatric clinic visits^2^ making it a worldwide public health issue^3^. The incidence of constipation in United States children is approximately 10% reported variously in different regions and based on different diagnostic criteria^2,4^. Functional constipation is the most common type, and an organic causes are less commonly recognized^5^.

Functional constipation is usually diagnosed based on history and physical examination^6^. In some cases when functional constipation is not likely, further investigation is necessary to rule out organic causes. This may be an expensive process^7^. Laboratory and radiographic testing and even rectal biopsy is performed for definite diagnosis of organic constipation^8^. Lead poisoning should be in the differential diagnosis of pediatric constipation diagnosed by blood lead level laboratory testing^9^.

Lead poisoning is often is caused by occupational or environmental exposures^10^. Children are more impacted by lead, especially neurodevelopmental complications. Children absorb more lead than adults. Due to hand-mouth behavior environmental exposures are common. Children have higher lead absorption rate^11^ which is augmented by iron or calcium deficiency. Neurological complications are permanent and irreversible making lead poisoning early detection more and more important in children^12^.

Screening for organic causes is rarely cost effective, with some recommend against screening^13^. There is not enough information regarding screening in outbreaks or regions with higher rates of lead poisoning. We aimed to evaluate BLL in pediatric and adolescent clinic visits due to constipation in Tehran, Iran during a sharp rise in lead poisoning. We also sought to find significant factors in patients’ history and presentation in the populations of normal and elevated BLL.

## Methods

This is a cross sectional study with prospective data collection. Data for the current project was gathered from pediatric and adolescent patients who visited in the gastroenterology clinics of Mofid Children’s Hospital and Loghman Hakim pediatric and pediatric gastroenterology clinic in Tehran, Iran. Patients younger than 18 years of age with the chief complaint of constipation were eligible for enrollment. All of the patients were outpatients. Constipation was defined according to Rome IV Criteria: the symptoms of functional constipation must include two or more of the following:

1. Straining more than 25% of defecations,
2. Lumpy or hard stools (BSFS type 1 or 2) more than 25% of defecations,
3. Sensation of incomplete evacuation more than one-fourth (25%) of defecations,
4. Sensation of anorectal obstruction/blockage more than one-fourth (25%) of defecations,
5. Manual maneuvers to facilitate more than one-fourth (25%) of defecations, and
6. Fewer than three spontaneous bowel movements per week^14^.

A predetermined questionnaire was used for data collection and was filled by the patients’ parents. The questionnaire included patients’ demographic data, their household situation including age of the buildings, any renovation or painting in the last 6 month, type of the wall paints and pipes used, type of the toys kids usually play with, presence of factories near to their places and living in industrial zones, history of playing with soil, employment status of the parents, history of drug addiction in the 1st degree family, vital signs, duration of the constipation, history for delayed meconium defecation, soiling, withholding, and Hirschsprung, abdominal and rectal exam results, presence of concurrent sign and symptoms, history of receiving treatment for constipation, history for the conduction of abdominal imaging, sonography, endoscopy, anorectal manometry, abdominal and intestinal surgeries, and the history for previous lead poisoning and treatment, complete blood count (CBC) results and BLL then were added to the questionnaires. The questionnaire was designed and endorsed by the whole study team which comprised of both expert pediatricians and medical toxicologists.

Blood samples were collected after questionnaire completion for CBC and measuring BLL after appropriate skin preparation with ethyl alcohol. BLL was measured using the atomic absorption technique by using the Lead care II device. The Magellan LeadCare II device assays BLL using an electrochemical technique (anodic stripping voltammetry) (Meridian Bioscience Cincinnati, Ohio, USA).

Statistical analysis of the data was analyzed using IBM SPSS 24th version. The Kolmogorov–Smirnoff test was applied to find the distribution pattern of quantitative variables. The Chi-square test was applied for finding the significant differences for qualitative variables. For the quantitative variables, the Mann Whitney U test and Student T-test were used for finding significant differences respectively for non-parametric and parametric variables. For multivariate analysis, binary logistic regression was used by entering the variables with P value ≤ 0.20 and having sufficient frequency in the equation. Significant findings were defined as P value ≤ 0.05.

### Ethics approval and consent to participate

All the procedures of the study follow the Helsinki declaration for human procedures and experiments. Before the enrolment of the subjects, the aim of the study and the procedure was described for patients’ parents, and signed informed consent was obtained for each patient for study participation. The feasibility of the study has been endorsed by the ethics committee of Shahid Beheshti University of medical sciences (Reference IR.SBMU.RETECH.REC.1397.494).

## Results

A total of 237 patients met the criteria for inclusion in the study. Of the total, 206 (86.92%) were visited in the gastroenterology clinic of Mofid Children’s Hospital, and remaining 31 (13.08%) were visited in the pediatric clinic of Loghman Hakim hospital. A slight majority (122; 51.48%) were female and 115 (48.52%) were male. The mean age of subjects was 7.72 years (± 3.73; range 3 months–18 years). The mean weight, height and BMI were respectively 27.70 ± 14.09, 122.60 ± 23.74, and 17.35 ± 4.27. Patients had constipation for an average of 59.31 ± 33.18 days on the day of visit. 49 (20.68%) patients had BLL ≥ 5 µg/dL. The mean for BLL in total population was 3.51 ± 3.67 µg/dL. Table 1 shows the data on the demographic data in the groups of patients with normal and elevated BLL and Table 2 has depicted results of complete blood count (CBC) test of the patients. None of the patients in neither of the groups had received any treatment for high blood lead levels before.

**Table 1:** Demographic data of the patients.

	Patients with normal BLL n = 188	Patients with BLL ≥ 5 µg/dL n = 49	P value
Age	7.90 ± 3.72	7.01 ± 3.70	0.147^a^
Gender	100 F (53.2%)	22 (44.9%)	0.301^a^
	88 (46.8%)	27 (55.1%)	
Weight (kg)	28.46 ± 14.51	24.71 ± 11.98	0.104^a^
Height (cm)	123.53 ± 24.12	118.98 ± 22.07	0.234^b^
BMI (kg/cm^2^)	17.53 ± 4.44	16.64 ± 3.46	0.481^a^

Duration of constipation was more than 2  months in both groups*.*

^a^Mann–Whitney U-test.

^b^Student’s T-test.

**Table 2:** Complete blood count (CBC) results of the patients.

	Patients with BLL ≥ 5 (n = 49)	Patients with normal BLL level (n = 188)	Total (n = 237)	P value^a^
WBC × 10^3^/µL				
Mean ± SD	8.86 ± 2.55	8.77 ± 2.83	8.79 ± 2.77	0.56
Min–max	3.90–16.80	3.80–23.00	3.80–23.00	
RBC × 10^6^/µL				
Mean ± SD	4.31 ± 0.66	4.25 ± 0.77	4.26 ± 0.75	0.42
Min–max	2.80–5.20	2.90–5.90	2.80–5.90	
HCT %				
Mean ± SD	37.34 ± 5.66	37.81 ± 6.42	37.71 ± 6.26	0.63
Min–max	30.00–54.00	25.00–58.00	25.00–58.00	
HGB g/dL				
Mean ± SD	11.46 ± 1.43	11.71 ± 1.49	11.66 ± 1.48	0.36
Min–max	8.40–1490	8.50–15.30	8.40–15.30	
MCV fL				
Mean ± SD	78.94 ± 7.41	77.98 ± 7.25	78.18 ± 7.28	0.50
Min–max	65 ± 100	54.00–91.00	54–100	
PLT × 10^3^/µL				
Mean ± SD	290.50 ± 104.97	281.10 ± 91.48	283.04 ± 94.27	0.71
Min–max	132–507	123–500	123–507	

^a^Mann–Whitney U-test.

Table 3 illustrates the data on the prevalence of the factors relating to patients’ history, sign and symptoms. Univariate analysis was applied for finding the factors having significant difference among patients having normal and elevated BLL (Table 3). As it can be seen, muscle pain (P value 0.02, OR 5.23, 95% CI 1.35–20.27), maternal educational status (P value 0.02, OR 2.30, 95% CI 1.16–4.54), and the history of endoscopic evaluation (P value 0.012, OR 2.74, 95% CI 1.18–6.36) were the factors which had significant differences (P value ˂ 0.05).

**Table 3:** Possible factors affecting the blood lead levels and symptoms/evaluations performed on the study groups (univariate analysis).

Variables	Patient with normal BLL	Patient with BLL ≥ 5 µg/dL	P value	Odd ratio (95% CI)
History of abdominal surgery	6 (5.7%)	0	Constant	
Drug addiction in parents	12 (6.4%)	3 (6.1%)	0.94	0.96 (0.26–3.53)
Previous treatment for constipation	52 (27.7%)	13 (26.5%)	0.87	0.94 (0.46–1.92)
Recent home renovation	40 (21.3%)	11 (22.4%)	0.86	1.07 (0.5–2.82)
Pica	2 (1.1%)	3 (6.1%)	0.05	6.24 (0.99–39.36)
Soil playing	40 (21.3%)	15 (30.6%)	0.17	1.63 (0.81–3.29)
Having special diet	9 (8.5%)	3 (10.7%)	0.71	1.29 (0.33–5.13)
Difficult child	6 (3.2%)	2 (4.1%)	0.76	1.29 (0.25–6.60)
Living in industrial zones	12 (6.4%)	4 (8.2%)	0.66	1.3 (0.4–4.22)
History of hirschsprung disease	2 (1.1%)	0	Constant	
History of intestinal surgeries	5 (2.7%)	2 (4.1%)	0.60	1.56 (0.29–8.28)
Delayed neonatal meconium passage	4 (2.1%)	0	Constant	1.27 (1.18–1.35)
Age (younger than 5 years)	55 (29.3%)	22 (44.9%)	0.19	0.78 (10.53–1.13)
Playing with metal toys	38 (20.2%)	10 (20.4%)	0.98	1.01 (0.46–2.21)
Playing with cotton toys	56 (29.8%)	19 (38.8%)	0.23	1.49 (0.78–2.87)
Playing with plastic toys	146 (77.7%)	37 (75.5%)	0.75	0.88 (0.42–1.85)
Abnormal abdominal imaging	17 (16%)	7 (25%)	0.27	1.74 (0.64–4.75)
Abnormal anorectal manometry	0	0	Constant	
History of reference to pediatric gastroenterologist	43 (39.4%)	16 (57.1%)	0.09	2.05 (0.88–4.75)
Metal pipes at home	120 (63.8%)	35 (71.4%)	0.32	1.42 (0.71–2.82)
Anemia	12 (6.4%)	2 (4.1%)	0.54	0.62 (0.13–2.87)
Black stool	19 (10.1%)	6 (12.2%)	0.66	1.24 (0.347–3.3)
Blowing	30 (16%)	4 (8.2%)	0.17	20.47 (0.16–1.4)
Bone pain	10 (5.3%)	3 (6.1%)	0.83	1.16 (0.31–4.39)
Diarrhea	7 (6.4%)	2 (7.1%)	0.89	1.12 (0.22–5.71)
Vomiting	26 (13.8)	9 (18.4%)	0.42	1.4(0.61–3.23)
Early puberty	0	0	Constant	
Pain in the extremities	10 (5.3%)	5 (10.2%)	0.21	2.02 (0.66–6.22)
Headache	17 (9%)	6 (12.2%)	0.50	1.40 (0.52–3.77)
Hearing/vision impairments	2 (1.1%)	1 (2%)	0.59	1.94 (0.17–21.8)
Heartburn	18 (9.6%)	4 (8.2%)	0.76	0.84 (0.27–2.6)
Lack of concentration	13 (6.9%)	2 (4.1%)	0.47	0.57 (0.12–2.63)
Late puberty	0	0	Constant	
Learning disabilities	8 (4.3%)	1 (2%)	0.47	0.47 (0.06–3.84)
Loss of appetite	48 (25.5%)	14 (28.6%)	0.67	1.17 (0.58–2.35)
Muscle pain	4 (2.1%)	5 (10.2%)	0.02	5.23 (1.35–20.27)
Muscle weakness	15 (8%)	5 (10.2%	0.62	1.31 (0.45–3.8)
Nausea	26 (13.8%)	9 (18.4%)	0.42	1.4 (0.61–3.23)
Obstipation	8 (4.3%)	2 (4.1%)	0.96	0.96 (0.2–4.66)
Paresthesia	2 (1.1%)	1 (2%)	0.59	1.94 (0.17–21.8)
soiling	2 (1.1%)	1 (2%)	0.58	1.94 (0.17–21.8)
Abnormal abdominal sonography	32 (30.2%)	9 (32.1%)	0.84	1.09 (0.45–2.68)
Previous endoscopic evaluation	24 (20.9)	13 (41.9%)	0.012	2.74 (1.18–6.36)
Herbal medicine consumption	5 (4.7%)	1 (3.6%)	0.79	0.75 (0.08–6.68)
Mother’s higher education level	98 (41.3%)	35 (14.8%)	0.02	2.3 (1.16–4.5)

Data on the multivariate analysis of the factors has been illustrated in Table 4. Muscle pain (P value 0.020, OR 24.74, 95% CI 1.67–365.83), positive history of performing diagnostic endoscopy (P value 0.024, OR 3.646, 95% CI 1.189–11.178), and educational status of the mothers (P value 0.020, OR 4.45, 95% CI 1.271–15.570) were the factors with significant differences. Cox and Snell R Square of the model was 0.213 and Nagelkerke R Square was 0.331.

**Table 4:** Possible factors affecting the blood lead levels and symptoms/evaluations performed on the study groups (multivariate binomial logistic regression analysis).

	P value	Exp (B)	95% CI (lower–upper)	
History of abdominal surgery	0.999	0.000	0.000	
Pica	0.070	9.43	0.835	106.620
Soil playing	0.852	1.121	0.338	3.724
Ice eating	0.291	2.709	0.426	17.233
Age (younger than 5)	0.128	0.636	0.354	1.140
Blowing	0.309	0.465	0.106	2.031
Muscle pain	0.020	24.737	1.673	365.827
Mothers’ education level	0.020	4.45	1.271	15.570
History of reference to pediatric gastroenterologist	0.404	1.527	0.565	4.126
Previous endoscopic evaluation	0.024	3.646	1.189	11.178

## Discussion

In the current study, nearly one-fifth of patients who presented with constipation had elevated BLL. Multivariate analysis found muscle pain, educational status of mothers, and history of performing diagnostic endoscopy for evaluation of chronic refractory abdominal pain as the factors showing significant differences in patients with elevated BLL.

Although preventive measures have been exerted to control lead exposure worldwide like the ban for use of lead in paints, lead poisoning has remained a challenge globally. Sources of lead may include old metal pipes, herbal medicine, old buildings and paints, imported toys, and candies^15,16^ in addition, outbreaks of lead poisoning have happened due to contaminated water^17^. In Iran, in recent years, a massive outbreak of lead poisoning occurred due to adulterated opium^18^. This fact especially aligns with the fact that the respiratory tract is the greatest source for absorbing lead^10^ and there are reports on lead poisoning due to inhaled opium^19^. Thus, opium addiction in the family may prone children to being exposed to lead by secondary inhaling or accidental ingestion of opium as it happened so^20^. Another source of lead poisoning has been air pollution^21^. In a study on pediatric lead poisoning in Tehran, the number of lead-poisoned children was relatively higher in industrial zones and in the districts which had the highest records of air pollution^22^. Tehran and other metropolises of Iran have been struggling with the problem of severe air pollution especially with particulate matter pollution in recent years and industrialization and lack of proper air pollution control plans have worsened the situation leading to a great undesirable impact on public health^23^. This pollution and related house dust also has been recognized as another source of lead poisoning^16^.

Lead poisoning can be presented with a variety of signs and symptoms including gastrointestinal manifestations including abdominal pain, constipation, anemia, fatigue, peripheral neuropathy, alteration in nervous system function, renal dysfunction, and in severe cases, papillary edema, coma, and convulsion^24^; yet, in many cases of lead exposure, it remains asymptomatic. These facts highlight the blood test for measuring BLL as the most definitive test to evaluate lead exposure as the center for disease control and prevention suggests^25^. The most concerning outcome of lead exposure in children is its permanent neurologic impairment and subsequent effects on concentration and learning abilities, which in turn influences the proper academic development of individuals and their achievements^26^. It is believed that children are more prone to neurologic complications in comparison to adults due to incomplete blood–brain barrier formation^27^. In the past, CDC was reporting only the cases of BLL ≥ 10 to parents but through years they reduce the level to 5^28^, and most recently they even lowered the level to 3.75 in the most recent recommendation^25^. However, it has been reported that lead exerts its deleterious effects on the neurologic and cognitive performance of individuals at any level^29^. These facts justify the utility of BLL measurement in children to prevent and lower lead exposure by proper interventions and in cases of BLL ≥ 45 with chelation therapy as CDC suggests^25^.

Constipation is one of the most common gastrointestinal manifestations of lead poisoning and it is believed to relate to the direct inhibitory effects of lead on the neural plexus of the intestine and also smooth muscles contractions. Metabolic changes are also involved like the accommodation of δ aminolaevulinic acid, a porphyrin precursor, due to porphyrinopathy induced by lead, which in turn lower the intestinal motility. Also, lead’s alteration in the excitation of the cell membrane through changes in the activity of cell membrane canals is effective on the occurrence of constipation^18^.

In the current study, history of upper endoscopy as a workup for chronic refractory abdominal discomfort showed a significant difference in patients with elevated BLL in comparison to patients having normal levels. Endoscopic work up was more than threefold higher in the group of patients with elevated BLL. It is recommended that endoscopic evaluation should be considered when constipation is followed by alarming signs including weight loss, abdominal pain or tenderness, lack of appetite, and gastrointestinal bleeding^30^. Since lead poisoning is compatible to manifest a variety of gastrointestinal symptoms in addition to constipation, it can be assumed this higher rate of endoscopy can be attributed to this fact. In another project on the presence of lead poisoning in adult patients with unjustified abdominal pain during a lead poisoning outbreak, endoscopic evaluation was also higher^18^. Thus, it may be beneficial to consider lead poisoning in the presence of gastrointestinal signs and symptoms especially during outbreaks and in the district with a high rate of lead contamination.

Also, there was a significant difference in the educational status of the mothers. This factor was also a significant factor in our former project on lead evaluation in pediatric patients with abdominal pain. We assumed that it is more probable that mothers with an academic degree got employed and housewives’ mothers are more involved in the cleaning of their living places. This fact is especially aligned with the finding that in both projects age (below 5) was a significant finding. This age is the period when children show hand-mouth behaviors and are more susceptible to hazardous consequences of lead exposure by ingesting sources of lead including house dust^22^. Also, it is said that a mother’s educational status is positively connected to child health and survival^31^. By this, it can be hypothesized that educated mothers may be more alerted to follow up the disease and alteration in the condition of their offspring and the real number of lead-exposed children may be higher.

Muscle pain was also another significant factor. Muscle pain and back pain also have been significant findings in painters and battery workers in a study conducted by Kuruvilla et al.^32^. In addition, Peterson (2010) reported a case of lead poisoning presenting with severe myalgia^33^. However, the mechanism by which lead induces muscle pain is not well documented.

### Limitations

This study has used questionnaire as the mean for gathering the data, thus, some of the questions remained unanswered and imposed missing data on the project. In addition, there was questions like the addiction history in the parents, that some parents may have been not totally accurate to answering them. Data was gathered in the clinic, and final diagnosis of patients remained unanswered, a follow up study could be useful to apply interventions to reduce lead exposure and to evaluate the consequences in constipation management of the patients. Another limitation was that the participants were not fully blinded to the objectives of this study. This fact might cause a potential bias. Moreover, food and water might contain certain amounts of lead, a fact that was not considered as an objective of this study and was therefore not evaluated.

## Conclusions

This study found a relatively high rate of elevated BLL among pediatric and adolescent patients seeking medical care for constipation. This finding may require future studies with a population size prospective on the screening for elevated BLL especially in susceptible groups and polluted districts. BLL testing and the possibility of lead poisoning can also eradicate the need for the invasive procedures like endoscopic evaluation especially regarding patients’ history and symptoms.

## Data Availability

The data that support the findings of this study are available from corresponding author but restrictions apply to the availability of these data, which were used under license for the current study, and so are not publicly available. Data are however available from the authors upon reasonable request and with permission of the “Iranian Registry of Pediatric Lead Poisoning” (IRPLP), Shahid Beheshti University of Medical Sciences.

## Abbreviations

BLL: Blood lead level
BMI: Bone marrow index
CBC: Complete blood count
CDC: Center for disease control
CI: Confidence interval
OR: Odds ratio
SPSS: Statistical package for the social sciences
